# 101 Surveillance and Mitigation of Hospital-Onset Respiratory Viral Illness Transmissions in Inpatient Psychiatry

**DOI:** 10.1017/ash.2026.10718

**Published:** 2026-06-23

**Authors:** Michelle Meyer, Emily Loberg, Sarah Bellows Mahler, Sheila Higbe, Vickie L. Miller, Elena Beam, Patricia Simner, Jennifer Lu, Robin Patel, Priya Sampathkumar

**Affiliations:** 1 Mayo Clinic; 2 Mayo Clinic Rochester; 3 Johns Hopkins Hospital; 4 Mayo Clinic, Rochester, MN

## Abstract

**Background:** We describe an investigation of a cluster of eight Corynebacterium amycolatum sternal surgical site infections (SSIs) that occurred among cardiac surgery patients from February to October 2025. Although C. amycolatum is a member of the skin microbiota, clinically significant infections caused by this organism may occur in cardiac surgery patients. **Methods:** The investigation included retrospective reviews of SSIs, clinical cultures and medical records, surgical observations, and operating room (OR) assessment. Line lists of surgical case criteria and infection prevention practices were created to assess common factors among cases (e.g., staffing, instrumentation, equipment, skin preparation and antisepsis, nasal decolonization, antibiotic protocols, and closure). Whole genome sequencing of isolates was performed and analyzed using SNIPPY for single-nucleotide polymorphism (SNP)-based phylogenetic analysis. **Results:** Phylogenetic analysis indicated isolates from five of eight case patients who had cardiac surgeries occurring over a six-week period from February to April 2025 were genetically related (?7 SNPs across the genome), suggesting a common source of infection. These five isolates were ceftriaxone, meropenem, and penicillin resistant. The other three case patient isolates were unrelated (<3181 to <15,000 SNPs); two were ceftriaxone, meropenem and penicillin resistant (one also doxycycline intermediate resistant), and the third penicillin resistant. Use of sternal tape for closure was identified as a recent practice change. Four of five genetically related cases involved the same surgeons and suture tape for sternal closure (wire for all other cases). Sternal tape was discontinued with three cases occurring afterwards. Seven patients completed chlorhexidine gluconate bathing and nasal decolonization. Hair was removed in the OR with reusable surgical clippers. All received the same skin antiseptic, and antibiotic prophylaxis was appropriate. No trends were identified in instrument or equipment use. Broad infection control measures were implemented, including surgical clipper replacement, OR cleaning and ultraviolet light disinfection, headlamp cleaning, and environmental repairs. Leadership was engaged through frequent meetings and audits. Infection control practices such as minimizing OR traffic, double gloving, glove changes, and strict adherence to hand hygiene and attire were reinforced. Two of eight cases occurred after these recommendations were completed but were not genetically related to the primary cluster. **Conclusion:** We describe a cluster of invasive C. amycolatum sternotomy SSIs. Only two genetically unrelated cases of C. amycolatum occurred following infection control investigation and interventions, underscoring the effectiveness of a comprehensive, multidisciplinary approach. This organism has rarely been reported as a cause of SSIs and may be an emerging pathogen.

